# Evidence for protein leverage in a general population sample of children and adolescents

**DOI:** 10.1038/s41430-023-01276-w

**Published:** 2023-02-16

**Authors:** Christoph Saner, Alistair M. Senior, Hanyue Zhang, Aino-Maija Eloranta, Costan G. Magnussen, Matthew A. Sabin, Markus Juonala, Marco Janner, David P. Burgner, Ursula Schwab, Eero A. Haapala, Berit L. Heitmann, Stephen J. Simpson, David Raubenheimer, Timo A. Lakka

**Affiliations:** 1 Division of Pediatric Endocrinology, Diabetology and Metabolism, Department of Pediatrics, Inselspital, Bern University Hospital, University of Bern, Bern, Switzerland; 2 Department of Biomedical Research, University of Bern, Bern, Switzerland; 3 Murdoch Children’s Research Institute, The Royal Children’s Hospital, Parkville, Victoria Australia; 4 Charles Perkins Centre and School of Life & Environmental Science, University of Sydney, Sydney, New South Wales Australia; 5 Research Unit for Dietary Studies at the Parker Institute, Bispebjerg and Frederiksberg Hospital, The Capital Region, Frederiksberg, Denmark; 6 Department of Public Health, Section for General Practice, University of Copenhagen, Copenhagen, Denmark; 7 Institute of Public Health and Clinical Nutrition, School of Medicine, University of Eastern Finland, Kuopio, Finland; 8 Institute of Biomedicine, School of Medicine, University of Eastern Finland, Kuopio, Finland; 9 Department of Medicine, Endocrinology and Clinical Nutrition, Kuopio University Hospital, Kuopio, Finland; 10 Baker Heart and Diabetes Institute, Melbourne, Victoria Australia; 11 Research Centre of Applied and Preventive Cardiovascular Medicine, University of Turku, Turku, Finland; 12 Centre for Population Health Research, University of Turku and Turku University Hospital, Turku, Finland; 13 Department of Endocrinology, The Royal Children’s Hospital, Parkville, Victoria Australia; 14 Department of Medicine, University of Turku, Turku, Finland; 15 Division of Medicine, Turku University Hospital, Turku, Finland; 16 Faculty of Sport and Health Sciences, University of Jyväskylä, Jyväskylä, Finland; 17 Department of Clinical Physiology and Nuclear Medicine, Kuopio University Hospital, University of Eastern Finland, Kuopio, Finland; 18 Foundation for Research in Health Exercise and Nutrition, Kuopio Research Institute of Exercise Medicine, Kuopio, Finland

**Keywords:** Epidemiology, Nutrition

## Abstract

**Background/Objectives:**

The strong regulation of protein intake can lead to overconsumption of total energy on diets with a low proportion of energy from protein, a process referred to as protein leverage. The protein leverage hypothesis posits that protein leverage explains variation in energy intake and potentially obesity in ecological settings. Here, we tested for protein leverage and the protein leverage hypothesis in children and adolescents.

**Subjects/Methods:**

A population sample of children, mean (SD) age 7.6 (0.4) years (*n* = 422), followed up at age 9.8 (0.4) years (*n* = 387) and at age 15.8 (0.4) years (*n* = 229), participating for the Physical Activity and Nutrition in Children (PANIC) study. Exposures: 4-day food records-related proportional energy intake of proteins, fats, and carbohydrates. Outcomes: energy intake, body mass index (BMI) z-score and dual-energy X-ray absorptiometry-related energy expenditure.

**Results:**

Proportional energy intake of proteins was inversely associated with energy intake following power functions at all 3 ages (mean [95%CI] strength of leverage of *L* = −0.36 [−0.47 to −0.25]; *L* = −0.26 [−0.37 to −0.15]; *L* = −0.25 [−0.38 to −0.13]; all *P* < 0.001). Mixture analysis indicated that variance in energy intake was associated primarily with the proportional intake of energy from proteins, not with either fats or carbohydrates. At all 3 ages, energy intake was not associated with BMI z-score but positively associated with energy expenditure (all *P* < 0.001).

**Conclusions:**

This study provides evidence consistent with protein leverage in a population sample of children and adolescents. Increased energy intake on diets with lower protein content was counterbalanced by increased energy expenditure and therefore did not translate into increased adiposity.

## Introduction

Overweight and obesity in childhood are major determinants of global health [1]. Excessive body mass index (BMI) in children and adolescents is associated with adverse psychosocial [2] and cardiometabolic health [3]. Children with obesity are at high risk of becoming adults with obesity [4], at increased risk of developing non-communicable diseases [5, 6], and at increased risk of premature mortality [7]. Overweight and obesity are caused by excessive total energy intake (TEI) at a given total energy expenditure (TEE), a physical law that is modulated by the individual’s genetic and epigenetic background and by adipose tissue-related alterations of control mechanisms for TEI and TEE [8].

In humans and organisms across many taxa, the interplay between specific appetite systems for carbohydrates, fats and proteins determine TEI. Exposed to a healthy food environment, these appetite systems will regulate food intake to meet individual macronutrient targets [9, 10]. However, when exposed to an unbalanced food environment, such as in diets with high amounts of ultra-processed foods [11], the competing appetite systems may result in a food intake that is determined by the relative strength of different macronutrient appetites [12].

Using the nutritional geometry framework, studies in animals [13, 14] and randomized control trials in adult humans [15] have shown that the intake of proteins is more strongly regulated than the intake of carbohydrates and fats. Consequently, on lower protein diets, energy is over-consumed as an inadvertent result of compensating for protein dilution, a mechanism referred to as protein leverage (PL), leading to increased risk of weight gain. The protein leverage hypothesis (PLH) posits that the PL mechanism interacts with dilution of dietary protein to drive energy over-consumption and risk of obesity in ecological settings [16].

Despite evidence for PL and PLH in adults, there are no published reports on PL and PLH in population samples of children and adolescents, and only one cross-sectional study providing evidence for PL and PLH in children and adolescents, from an Australian cohort with severe obesity [17]. There are no published reports on PL and PLH in population samples of children and adolescents. Here, we first tested for PL in a population sample of Finnish children starting first grade primary school, who were followed up until adolescence. Second, we tested whether TEI on lower dietary protein diets was associated with adiposity, assessed by measures of body size and composition.

## Methods

### Study design and participants

The present study is based on data from participants of the Physical Activity and Nutrition in Children (PANIC) study. The PANIC study is an 8-year, single-center, controlled trial on the effects of a combined physical activity and dietary intervention on health outcomes in a general population sample of children from Kuopio, Finland (http://www.panicstudy.fi). Data were collected in individuals aged 6–8 years (T0), at the 2-year follow-up (T1) and at the 8-year follow-up (T2). The Research Ethics Committee of the Hospital District of Northern Savo approved the study protocol in 2006 (Statement 69/2006). The parents or caregivers of the children gave their written informed consent, and the children provided their assent to participation. The PANIC study has been carried out in accordance with the principles of the Declaration of Helsinki as revised in 2008 (see Supplementary Methods for further methodological details).

### Assessment of body size, body composition and Tanner stage

At all ages, weight (kg), height (m), body size measures (waist circumference, WC in cm; BMI in kg/m^2^) and BMI *z*-score using Finnish reference growth charts [18]) and body composition measures (total and %fat and %lean mass by dual-energy X-ray absorptiometry) were assessed. Pubertal stage was evaluated by a trained physician according to Tanner’s classification, where pubertal onset is determined by breast development in girls [19] and a testes volume ≥4 ml in boys [20].

### Assessment of nutrition, physical activity and total energy expenditure

Energy and nutrient intake was assessed from consecutive 4-day food records [21]. Total energy intake (kcal) as well as absolute (in g) and proportional energy from proteins (%EP), carbohydrates (%EC) and fats (%EF) were calculated using the Micro Nutrica^®^ dietary analyses software, Version 2.5. Physical activity and sedentary time were assessed by questionnaires, and by a combined heart rate and body movement sensor (Actiheart^®^, CamNtech Ltd., Papworth, UK) (see Supplementary Methods).

TEE (in kilocalories) was calculated following *Pontzer* et al. [22] based on LM and FM assessed by dual-energy X-ray absorptiometry, according to the following formula [22]:

$$\ln \left( {TEE} \right) = - 0.121 + 0.696 \ast \ln \left( {LM} \right) - 0.041 \ast \ln \left( {FM} \right)$$

### Statistical analysis

Descriptive statistics are presented as means and standard deviation (SD) values for continuous variables and absolute numbers and percentages for categorical variables. Student’s *t* tests were used to compare continuous variables between participants in the intervention group and those in the control group at T0, T1 and T2. Associations between TEI and adiposity measures (BMI *z*-score, WC, %LM and %BF) and TEE were analyzed using multiple linear regression and adjusted for age and sex. Regression results were given as standardized regression coefficients β, their 95% confidence intervals (95% CIs) and their *P* values.

Protein leverage is indicated when there is a negative relationship between %EP and TEI. Were absolute protein intake regulated to a fixed intake (i.e., complete PL), the form of that relationship would be a power function, TEI = P × p^*L*^ [9, 23], where P is the target (regulated) intake (kcal) of protein and p is the proportion of protein in the diet. Partial PL is indicated when the exponent in the equation is >−1 but <0 (indicating no leverage). Human studies to date have yielded values for the exponent (*L*) in the range −0.3 to −0.6 [9]. The power function above was fitted as a linear regression such that log(TEI) = log(P) + log(p) × *L*. Values for TEI and p were derived from a nutrient assessment at each age. Models were adjusted for potential confounders of the association between %EP and TEI, including fiber intake, physical activity, sedentary time, age and TEE [9]. Variables used for adjusted models were normalized (i.e., scaled to zero) for comparison of their individual impact.

Where PL was detected, we tested for interactive or additive effects by sex (male versus female), by pubertal stage (pre-pubertal versus pubertal at T0 and T1) and by study group (intervention versus control) in sub-analyses. Each categorical variable was tested for an interactive effect with %EP (i.e., the significant *P* value for the interaction term in the power function), or for a significant (additive) effect when added to the power model.

A single nutrient association with TEI does not, however, account for the inevitable covariances between nutrients within dietary mixtures. Hence, a negative association between %EP and TEI will be accompanied by a positive association between some combination of %EC and %EF, leaving unresolved whether increased energy intake is driven by protein leverage or some quality of fat and carbohydrates. However, these alternatives were tested by using mixture modeling as explained in detail by the framework of nutritional geometry [24]. In brief, mixture models are used to analyze the association between the composition and interactions of macronutrients on TEI with the “MixModel” function in the R package mixexp [25]. In mixture model analysis, a total of 5 models with increasing complexity are built and compared against a null model (Model 1), which infers no effect of dietary composition on TEI. Model selection is based on the Akaike information criterion (AIC) [26], whereby the lowest AIC values are preferred. If two models were within two AIC points of each other, we chose the simplest model.

Adjustments for normalized data on fiber intake, age, TEE and questionnaire-related physical activity and sedentary time as potential confounders were made using linear mixture models. For visual interpretation, we illustrated the estimated associations from adjusted mixture models in right-angled mixture triangles with the proportional energy intake from proteins (*x*-axis), carbohydrates (*y*-axis) and fats (implicit axis indicated by distance to the hypotenuse) as predictors and a colored response surface for TEI [27]. The fitted coefficients for individual macronutrients, their interactions and covariates are provided as Supplementary Tables.

Axiomatically, for PL-related higher TEI to be associated with higher adiposity, TEI should be positively associated with the relevant adiposity measure. Therefore, mixture model analyses were performed for those adiposity measure that were positively associated with TEI as per adjusted linear regression models.

All statistical analysis were performed using R Studio, Version 1.1.453 [28]. A 2-tailed test with *P* < 0.05 was considered statistically significant.

## Results

From the 506 participants of the PANIC study, a total of 422 children (217 males) with a mean (SD) age of 7.6 (0.4) years had data at T0 (baseline). Of those, a total of 387 individuals (184 females) had follow-up data at T1 when aged 9.8 (0.4) years, and a total of 229 individuals (114 females) had follow-up data at T2 when aged 15.8 (0.4) years. The BMI z-score was −0.19 (1.08), −0.13 (1.07) and −0.03 (0.99) at the three ages, respectively. At T0, only 12 individuals (2.9%, 9 girls and 3 boys) were pubertal, whereas all participants were pubertal at T2. TEI increased with age from 1620 (303) kcal at T0 to 1820 (539) kcal at T2, as did TEE from 1660 (126) kcal at T0 to 2730 (385) kcal at T2 (Table 1).

**Table 1:** Cohort characteristics.

	Baseline, T0 *n* = 422	Follow-up 1, T1 *n* = 387	Follow-up 2, T2 *n* = 229
Sex			
Female	205 (48.6%)	184 (47.5%)	114 (49.8%)
Male	217 (51.4%)	203 (52.5%)	115 (50.2%)
Age (years)	7.6 (0.4, 6.8–9.0)	9.8 (0.4, 8.8–11.2)	15.8 (0.4, 15.0–17.4)
Weight (kg)	26.9 (4.99, 15.2–51.4)	34.4 (7.43, 18.0–68.7)	61.8 (13.4, 39.2–151)
Height (cm)	129 (5.6, 111–145)	140 (6.4, 121–161)	171 (8.5, 148–195)
Body Mass Index (kg/m^2^)	16.1 (2.1, 12.4–25.3)	17.3 (2.7, 12.3–27.7)	21.0 (3.5, 14.9–40.1)
Body Mass Index SD	−0.19 (1.08, −3.54–2.56)	−0.13 (1.07, −3.28–2.34)	−0.03 (0.99, −3.01–2.81)
Waist circumference (cm)	56.7 (5.9, 43.5–88.0)	61.4 (7.4, 42.2–95.4)	73.0 (9.3, 58.5–132)
Puberty	*n* = 420	*n* = 372	*n* = 229
Prepubertal	408 (97.1%)	286 (76.9%)	0
Pubertal	12 (2.9%)	86 (23.1%)	229 (100%)
Dual X-Ray Absorptiometry (DXA)	*n* = 414	*n* = 372	*n* = 227
% Lean mass	76.7 (8.0, 51.5–90.8)	72.9 (9.1, 45.9–89.6)	72.9 (9.8, 46.5–90.5)
Lean mass (kg)	20.7 (2.4, 13.3–28.1)	24.8 (3.2, 15.7–35.8)	44.6 (8.7, 26.3–68.5)
% Body fat	19.7 (8.2, 5.4–45.4)	23.4 (9.3, 6.7–50.8)	22.9 (9.9, 4.97–50.1)
Fat mass (kg)	5.7 (3.5, 1.3–22.6)	8.7 (5.3, 1.6–33.5)	14.5 (8.4, 2.7–58.7)
Total energy expenditure (kcal)	1660 (126, 1290–2050)	1860 (150, 1440–2270)	2730 (385, 1870–3630)
Self-reported physical activity (PA)	*n* = 422	*n* = 385	*n* = 228
Total physical activity (min)	112 (41.7, 31.4–247)	115 (42.8, 27.1–231)	144 (105, 13.5–835)
Total sedentary time (min)	217 (106, 19.3–856)	222 (93.7, 17.1–745)	516 (243, 57.9–1390)
Accelerometery-related PA	*n* = 381	*n* = 331	*n* = 122
Light PA (>1.5 to ≤4 METs in min)	508 (105, 151–767)	403 (87.5, 210–684)	324 (111, 105–691)
Moderate PA (>4 to ≤7 METs in min)	92.6 (53.9, 1.36–301)	75.6 (40.1, 4.84–220)	35.2 (25.7, 2.08–119)
Vigorous PA (>7 METs in min)	23.1 (23.0, 0–132)	25.7 (25.5, 0–132)	11.5 (14.7, 0–77.8)
Total PA (LPA, MPA, VPA in min)	624 (130, 169–905)	505 (107, 215–800)	371 (132, 126–760)
Sedentary time including sleep (≤1.5 METs in min)	814 (131, 519–1270)	933 (108, 613–1220)	1070 (133, 680–1310)
Sedentary time excluding sleep (≤1.5 METs in min)	233 (128, −41.8–666)	383 (104, 132–647)	604 (138, 223–918)
Dietary characteristics	*n* = 422	*n* = 387	*n* = 229
Total energy intake, TEI (kcal)	1620 (303, 706–2560)	1670 (341, 720–2610)	1820 (539, 800–4010)
Carbohydrates			
Absolute intake (g)	213 (44.7, 104–390)	213 (46.9, 77.2–366)	216 (72.0, 86.1–441)
Intake per kgBW	8.10 (1.99, 3.54–14.5)	6.43 (1.82, 2.12–13.3)	3.60 (1.27, 1.11–7.85)
Proportional intake (%/TEI)	52.6 (5.1, 36.8–67.9)	51.1 (5.2, 34.8–67.1)	47.6 (7.2, 26.0–67.0)
Fats			
Absolute intake (g)	55.0 (14.8, 19.8–105)	59.3 (17.0, 21.4–122)	70.1 (25.3, 22.4–160)
Intake per kgBW	2.10 (0.65, 0.69–4.41)	1.79 (0.60, 0.57–4.70)	1.17 (0.44, 0.37–2.75)
Proportional intake (%/TEI)	30.4 (5.0, 17.3–48.5)	31.8 (5.3, 16.0–48.2)	34.6 (6.6, 15.0–56.3)
Proteins			
Absolute intake (g)	68.5 (14.5, 28.5–114)	70.7 (16.6, 30.8–121)	80.5 (29.4, 30.8–224)
Intake per kgBW	2.60 (0.64, 1.13–4.88)	2.13 (0.60, 0.78–4.28)	1.33 (0.48, 0.47–3.08)
Proportional intake (%/TEI)	17.0 (2.5, 9.6–25.8)	17.0 (2.6, 7.2–26.1)	17.8 (3.8, 7.7–37.8)

Data from continuous variables are presented as mean (standard deviation, SD and range, minimum-maximum value), categorical variables are presented as total number (percentage, %).

*N* number of individuals, *SD* standard deviation, *MET* metabolic equivalents of task where one equivalent corresponds to 3.5 mL O2/kg/min (i.e., 71.225 J/kg/min), *LPA* light physical activity, *MPA* moderate physical activity, *VPA* vigorous physical activity, *TEI* total energy intake, *g* gram, *kgBW* kilogram bodyweight.

In linear regression models, significant associations were not found between TEI and either BMI *z*-score or WC at any age, nor between TEI with %LM or %BF at T0 and T1. At T2, TEI was directly associated with %LM, but inversely associated with %BF. In contrast, TEI was directly associated with TEE at all three ages (Supplementary Table 1).

### Power model analysis for proportional macronutrient intake toward total energy intake

**Fig. 1:**
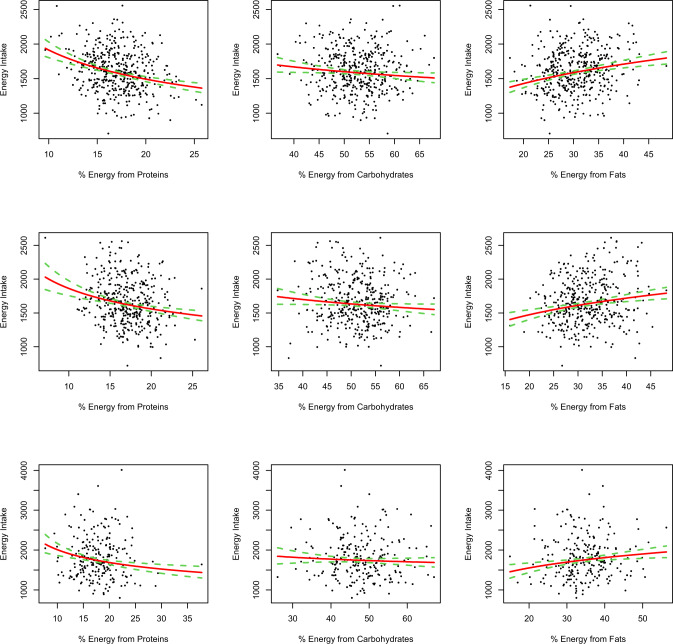
Adjusted power functions between proportional macronutrient intake and total energy intake. Adjusted power functions between proportional energy intake from macronutrients in relation to total energy intake at T0, 8 years (top array, *n* = 414), at T1, 10 years (middle array, *n* = 370) and at T2, 16 years (lowest array, *n* = 226). Red line, fitted mean; green dashed line, 95% confidence interval of fitted mean.

In power-models adjusted for dietary fiber, age, TEE and questionnaire-related physical activity and sedentary time, the %EP, was inversely associated with TEI, following a power function with strengths of leverage *L* of −0.36 at T0 (*P* < 0.001), *L* of −0.26 at T1 (*P* < 0.001) and *L* of −0.25 at T2 (*P* < 0.001). The %EC was also inversely associated with TEI at T0 with *L* = −0.19 (*P* = 0.029) and T1 with *L* = −0.17 (*P* = 0.045), but less strongly than protein, and there was no association at T2. The %EF was positively associated with TEI at all three ages with *L* = 0.26 at T0 (*P* < 0.001), *L* = 0.22 at T1 (*p* < 0.001) and *L* = 0.22 at T2 (*P* = 0.002) (Fig. 1 and Table 2).

**Table 2:** Model characteristics of adjusted power functions between proportional macronutrient and total energy intake.

*Predictors*	*Estimate*	*95% CI*	*P*	*Predictors*	*Estimate*	*95% CI*	*P*	*Predictors*	*Estimate*	*95% CI*	*P*
Log energy intake at T0, *n* = 414											
Log % E from Proteins	−0.36	−0.47 to −0.25	**<0.001**	Log % E from Carbohydrates	−0.19	−0.36 to −0.02	**0.029**	Log % E from Fats	0.26	0.16–0.36	**<0.001**
Fiber intake (SD)	0.07	0.05–0.08	**<0.001**	Fiber intake (SD)	0.07	0.05–0.08	**<0.001**	Fiber intake (SD)	0.07	0.05–0.09	**<0.001**
Physical activity (SD)	−0.00	−0.02–0.02	0.94	Physical activity (SD)	0.01	−0.01–0.03	0.37	Physical activity (SD)	0.01	−0.01–0.02	0.42
Sedentary time (SD)	−0.00	−0.02–0.01	0.94	Sedentary time (SD)	0.00	−0.02–0.02	0.89	Sedentary time (SD)	0.00	−0.02–0.02	0.93
Age (SD)	−0.01	−0.03–0.01	0.30	Age (SD)	0.00	−0.02–0.01	0.63	Age (SD)	−0.01	−0.03–0.01	0.35
Total energy expenditure (SD)	0.06	0.04–0.08	**<0.001**	Total energy expenditure (SD)	0.05	0.04–0.07	**<0.001**	Total energy expenditure (SD)	0.06	0.04–0.07	**<0.001**
**Log energy intake at T1,** ***n*** = 372											
Log % E from Proteins	−0.26	−0.37 to −0.15	**<0.001**	Log % E from Carbohydrates	−0.17	−0.35 to −0.00	**0.045**	Log % E from Fats	0.22	0.12–0.33	**<0.001**
Fiber intake (SD)	0.09	0.08–0.11	**<0.001**	Fiber intake (SD)	0.09	0.08–0.11	**<0.001**	Fiber intake (SD)	0.09	0.08–0.11	**<0.001**
Physical activity (SD)	0.00	−0.01–0.02	0.70	Physical activity (SD)	−0.00	−0.02–0.01	0.61	Physical activity (SD)	−0.00	−0.02–0.01	0.67
Sedentary time (SD)	0.01	−0.01–0.03	0.17	Sedentary time (SD)	0.01	−0.01–0.03	0.26	Sedentary time (SD)	0.01	−0.01–0.03	0.25
Age (SD)	−0.01	−0.03–0.01	0.19	Age (SD)	−0.01	−0.03–0.01	0.25	Age (SD)	−0.01	−0.03–0.00	0.14
Total energy expenditure (SD)	0.06	0.04–0.07	**<0.001**	Total energy expenditure (SD)	0.05	0.04–0.07	**<0.001**	Total energy expenditure (SD)	0.06	0.04–0.07	**<0.001**
***Log energy intake at T2, n*** ***=*** ***226***											
Log % E from Proteins	−0.25	−0.38 to −0.13	**<0.001**	Log % E from Carbohydrates	−0. 09	−0.27–0.09	0.31	Log % E from Fats	0.22	0.08–0.36	**0.002**
Fiber intake (SD)	0.15	0.12–0.17	**<0.001**	Fiber intake (SD)	0.15	0.12–0.18	**<0.001**	Fiber intake (SD)	0.15	0.12–0.18	**<0.001**
Physical activity (SD)	−0.03	−0.06–0.01	0.12	Physical activity (SD)	−0.03	−0.07 to −0.00	**0.045**	Physical activity (SD)	−0.03	−0.07–0.00	**0.039**
Sedentary time (SD)	−0.02	−0.05–0.01	0.13	Sedentary time (SD)	−0.02	−0.05–0.01	0.18	Sedentary time (SD)	−0.03	−0.05–0.00	0.09
Age (SD)	−0.01	−0.03–0.02	0.64	Age (SD)	−0.01	−0.04–0.02	0.48	Age (SD)	−0.01	−0.04–0.02	0.42
Total energy expenditure (SD)	0.13	0.10–0.16	**<0.001**	Total energy expenditure (SD)	0.12	0.09–0.15	**<0.001**	Total energy expenditure (SD)	0.12	0.09–0.15	**<0.001**

Adjusted power analysis results for log % Energy from proteins (left column), carbohydrates (middle column) and fats (right column) in relation to log total energy intake. Results at To, 8 years in top array, at T1, 10 years in middle array and at T2, 16 years in lowest array. Models adjusted for normalized intake of fiber (in g), for physical activity and sedentary time (in min), age (in years) and total energy expenditure (in kcal).

*n* number of individuals, *95%CI* 95% confidence interval, *P P* value, *SD* standard deviation, *E* energy in kcal.

Bold *P* values indicate a level of significance <0.05.

### Linear mixture model results between macronutrient composition with total energy intake

**Fig. 2:**
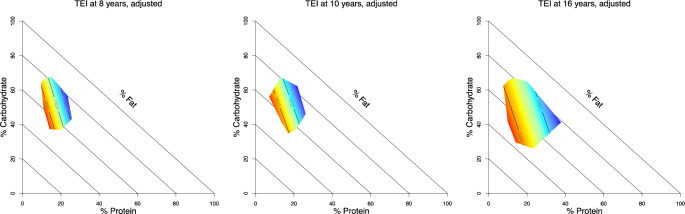
Right-angled mixture triangles for total energy intake, adjusted. Adjusted response surface from linear mixture models from proportional intake of proteins (*x*-axis), carbohydrates (*y*-axis) and fats (implicit axis) as compositional predictors and total energy intake as outcome. A red surface indicates a higher outcome level, a blue surface indicates a lower outcome measure. TEI: total energy intake (kcal).

The interactions between %EP, %EC and %EF were disentangled using mixture modelling, which showed that %EP was primarily and inversely associated with TEI in adjusted linear mixture models. This is illustrated by the shape of the TEI response surface in right-angled mixture triangles (Fig. 2), where the principal gradient for TEI follows the percent protein axis (i.e., isolines on the response surface are near vertical). The fitted coefficients are given in Supplementary Table 2.

### Sub-analysis to test for impact of sex, pubertal onset, study group, accelerometry-related physical activity and underreporting of energy intake with respect to protein leverage

Male sex was a significant, additive covariate in adjusted power-models and associated with higher TEI at all ages compared to females (Supplementary Fig. 1 and Supplementary Table 3). Neither pubertal status nor study group (intervention versus control) was associated with TEI in adjusted power-models (neither as an interactive term with %EP nor as an additive covariate, data not shown). Differences in study groups (intervention versus controls) are summarized in Supplementary Results.

In power-models adjusted for Actiheart-assessed physical activity and sedentary time, %EP was inversely associated with TEI with strengths of leverage *L* at T0, T1 and T2 comparable to data adjusted for questionnaire-assessed physical activity and sedentary time (Supplementary Table 4).

A total of 191 (45%) individuals at T0, 108 (28%) individuals at T1 and 7 (3%) individuals at T2 had a higher reported TEI than their TEE. However, irrespective of whether the reported TEI exceeded or fell below TEE, the results from linear mixture models of macronutrient composition with TEI were similar to the whole cohort (data not shown).

## Discussion

This is the first study to provide evidence for PL in a population sample of children and adolescents. In subgroup analyses, male sex was positively associated with TEI, whereas pubertal stage and participation in lifestyle intervention were not associated with TEI. Despite evidence for PL at each of the 3 ages, we did not find a significant relationship between total energy intake and BMI *z*-score or WC, most likely explained by countermanding changes in TEE.

This counterbalancing of TEI by TEE is suggestive that the population was in energy balance. Consistent with this suggestion was that, according to Finnish reference data, the BMI *z*-scores at all 3 ages were close to zero and the prevalence of overweight and obesity in this cohort of children aged 6.8–9.0 years was only 13.1% (obesity 4.3%), which is low compared to corresponding international data [29, 30].The second reason for no association between TEI and higher adiposity might be underreporting of TEI in dietary assessments, which is well-known for certain subgroups of the population, such as adolescents and those with obesity [31]. In fact, the ratio of TEE/TEI was greater than 1 (1.02 at 8 years, 1.11 at age 10 years and 1.5 at age 16 years), indicative of underreporting of TEI. However, if TEI had been consistently higher than reported, we would have expected the prevalence of overweight and obesity in PANIC to have been higher than it was.

Third, this cohort of children and adolescents had a relatively healthy diet. Some evidence for healthy eating habits in Finnish youth was shown in a recent study investigating a nationwide cohort of 10,569 Finnish children aged 9–14 years, where the proportion of unhealthy eaters was relatively low at 12.3% [32]. The effect of PL on TEI and potentially BMI *z*-score is expected to be most prominent when individuals are exposed to a food environment rich in highly processed diets, which are low in the proportion of proteins relative to energy-dense carbohydrates and fats and simultaneously low in fiber, which like protein is a satiating food component [9]. The effects of PL are also predicted to be exacerbated in populations in which the target for protein is elevated. Examples include populations undergoing a nutrition transition from traditional, high-protein diets (for Inuit a %EP in excess of 30% [33]) to a Westernized diet, with high rates of obesity and metabolic diseases as a consequence [34]. This is because the effects of PL are exacerbated on a given low-percent protein diet when the target intake is higher, due to the power function relating %TEI to %EP [22, 35]. Since protein targets also rise with obesity and insulin resistance as a result of increased protein catabolism and gluconeogenesis [9, 16], this may explain evidence for increased obesity on lower protein diets in children and adolescents with severe obesity [17].

How do our findings translate to children and adolescents living in modern industrialized food environments? The *Global Burden of Disease 2017* study involving data between 1990 and 2017 from 195 countries identified several dietary risk factors contributing to non-communicable disease morbidity and mortality [36]. These risk factors included diets high in sugar-sweetened beverages, high in sodium, high in processed meat (containing ~20% fat compared to ~5% in whole trimmed meat [37]), and low in nuts, seeds, vegetables, fruits, and fiber. A diet rich in ultra-processed foods incorporates most of these risk factors. Ultra-processed foods are ready-to-eat products made of processed substances, typically containing artificial flavors, colors, and cosmetic additives. They contain high amounts of refined chemicals, including carbohydrates and saturated fatty acids, low amounts of proteins and fiber, reflecting industrial remnants of whole foods [38, 39]. Over the last few decades, the sale and consumption of ultra-processed foods have increased globally [40]. In the United States (US), according to 24 h dietary recalls in 23,847 adult individuals, nearly 60% of calories consumed in the period between 2007 and 2012 came from ultra-processed foods [41]. Similar, 65% of calories consumed by primary and secondary school children in the United Kingdom (UK) are derived from ultra-processed foods [42]. Children and adolescents represent the largest group of consumers for ultra-processed foods in Canada and the US [41, 43]. Marketing campaigns are aimed at children [44] and eating while watching TV has been identified as a factor associated with higher consumption of ultra-processed foods in 1772 children aged 4–10 years in the UK [45]. From the present results indicating protein leverage, we predict that children and adolescents who are chronically exposed to a diet containing high quantities of ultra-processed foods will suffer the expected consequence of increased adiposity [16].

The normal range of pubertal onset for girls is between 8 and 13 years, and for boys between 9 and 14 years. Over the last two centuries, environmental changes including nutritional exposures have been considered responsible for a secular trend toward earlier pubertal onset [35, 46]. Consistent evidence exists for higher adiposity and earlier puberty in girls [47], with equivalent findings from observational studies in boys [48]. Our findings show higher TEI for boys at T0, T1 and T2 compared to girls, but no difference in BMI *z*-scores at T0, T1 or T2 between the sexes and no effect of pubertal stage on PL. Further longitudinal studies are required to confirm whether, and if in what direction, PL may directly or indirectly impact on puberty.

Strengths of this study include the comprehensive data collection at three ages on body size and composition, diet, and physical activity in a population sample, and the use of multi-dimensional nutritional models which consider dietary mixtures of macronutrients rather than effects from single macronutrients, which do not account for autocorrelations between nutrients within dietary mixtures [49]. Our results for PL in children and adolescents are novel and robust to adjustment for a number of significant potential confounders. A limitation of this study is the cross-sectional analysis. Studies with shorter intervals of data assessments are needed to investigate the predictive impact of macronutrient composition on changes in TEI. Second, our results were based on a population cohort of children and adolescents with a relatively low prevalence of overweight and obesity, living in a relatively healthy food environment, and therefore, warrant replication in other cohorts in different environments to assess generalizability. Third, the sub-analyses for the associations of PL with puberty were drawn from a lower number of participants and warrant further studies.

## Conclusion

This is the first study to provide evidence for PL in a population-representative cohort of children and adolescents. In this cohort, PL was counterbalanced by higher TEE and therefore, was not associated with adiposity, which we hypothesize to reflect having a predominantly healthy-weight cohort consuming a generally healthy diet.

## Supplementary information

Supplemental material

## Data Availability

Data analyzed in this study are available from the corresponding author on reasonable request.
