# Supplementary material for: Evidence for protein leverage in a general population sample of children and adolescents
Source: Eur J Clin Nutr. 2023 Feb 16;77(6):652–9. doi: 10.1038/s41430-023-01276-w (PMC10247372; doi:10.1038/s41430-023-01276-w)
Supplement: Supplementary file 1 — Supplemental material [file 41430_2023_1276_MOESM1_ESM.docx]

**Online-Only Supplementary Text**

**Supplementary Methods**

*Characteristics of the Physical Activity and Nutrition in Children (PANIC) intervention study*

At baseline between 2007-2009 (T0), a total of 736 children aged 6.8-9 years who started first grade in sixteen primary schools of a geographically and socioeconomically balanced area in the city of Kuopio were invited to participate. Exclusion criteria were physical disabilities, and no time or motivation to attend the study. A total of 512 (70%) children (248 girls, 264 boys) accepted participation, and after exclusion of 6 children due to physical disabilities or withdrawal of their families, a total of 506 children were allocated to an intervention and a control group after baseline examinations [1]. The intervention comprised of six individualized and family-based physical activity and dietary counselling sessions within the first two years. All children were invited to the 2-year follow-up examination in 2009-2011 (T1) when they were aged 9-11 years. The intervention group continued with seven physical activity and dietary counselling sessions until the 8-year follow-up examinations carried out in 2015-2017 (T2) when the participants were 15-17 years of age. In brief, the intervention goals were to increase the time spent on physical activity, to decrease sedentary time, to increase fiber and unsaturated fat intake and to avoid excessive energy intake and decrease the consumption of food containing large amounts of saturated fat, sugar, and salt [2].

*Assessment of body size and body composition*

Consecutive 4-day food records, filled out by parents (at T0 and T1) and adolescents (at T2), including at least 1 weekend day were used to assess energy and nutrient intake [3]. At T0, T1 and T2, weight was measured in light underwear to the nearest 0.1 kg using a calibrated InBody 720 ® bioelectrical impedance device (Biospace, Seoul, South Korea) with the weight assessment integrated into the system. Height was measured to the nearest 0.1 cm using a wall-mounted stadiometer. BMI was calculated by weight (in kg) divided by height (in meters squared, m^2^), and BMI z-scores were derived using Finnish reference growth charts [4]. Waist circumference was measured to the nearest 0.5 cm using a tape midway between the lower rib and upper iliac crest. Body composition was determined by total lean mass (LM), total fat mass (FM), and proportional (in percentage, %) lean mass (%LM) and body fat (%BF), measured using a Lunar® dual-energy X-ray absorptiometry (DXA) device (Lunar Prodigy Advance, GE Medical Systems, Madison, WI, USA) [5].

*Assessment of nutrition*

Validity of analogue food record methods against the observation method in preschool and primary school children is available [6, 7]. To improve the reliability of the food records, parents and adolescents were instructed by a clinical nutritionist on household and other measures to assess quantities and portion sizes were completed using a reference picture book [8]. Total energy and nutrient intakes were calculated using Micro Nutrica® dietary analyses software, Version 2.5. that utilizes Finnish and international data on nutrient contents of foods [9]. Net intake of proteins, carbohydrates, and fats in grams were multiplied by 4 (for proteins and carbohydrates) and 9 (for fats) to determine energy intake in kilocalories (kcal). The proportional energy intake of each macronutrient was calculated by the relevant macronutrient’s energy intake (kcal) divided by TEI (kcal) and multiplied by 100% to reveal percentage energy intake from proteins (%EP), carbohydrates (%EC) and fats (%EF).

*Assessment of physical activity and sedentary time*

Physical activity and sedentary time were assessed by the PANIC Physical Activity and Hobby Questionnaire, reported by parents (at T0 and T1) and adolescents (at T2) [22]. The types of physical activity included commuting to and from school, physical education at school, physical activity during recess, organized sports, competitions and games in sports, organized exercise other than sports, and unsupervised physical activity. The adolescents were asked to report whether they had done specific types of physical activity during the previous year. They were then asked to report the number of months of each physical activity per year, the number of sessions of physical activity per week, and the duration of a single session of each type of physical activity separately on weekdays and weekend days. The total amount of each physical activity was calculated and expressed in hours per day. The amount of total physical activity was calculated by summing the amount of each physical activity type and was expressed in hours per day. Sedentary time included watching TV and videos, using the computer or a tablet, playing video games, using a mobile phone and playing mobile games, listening to music, playing musical instruments, singing, reading, writing, drawing, doing arts and crafts, playing board and card games, cooking and baking, and sitting and lying for a rest. The amount of sedentary time was calculated by summing the amount of each type of sedentary time, weighted by the numbers of weekdays and weekend days, and was expressed in hours per day.

Also, a combined heart rate and body movement sensor (Actiheart®, CamNtech Ltd., Papworth, UK) with two standard electrocardiogram electrodes (Bio Protech Inc., Wonju, South Korea) was attached to the child`s chest and set to record in 60-s epochs. Non-wear was classified as >90 min periods of non-movement if accompanied by non-physiological heart rate and was accounted for when summarizing the time series as previously validated [23]. Participants were requested to wear the sensor for a minimum of four consecutive days without interruption, including two weekdays and two weekend days [24]. They were instructed to wear the monitor continuously, including sleep and water-based activities, and not to change their usual behavior during the monitoring period. The heart rate was pre-processed [25] and estimated physical activity intensity time-series using the individual calibration of heart rate combined with movement in a branched equation modelling framework were used [26, 27]. Physical activity was summarized as physical activity volume (J/kg/min) and time spent at specific intensity levels in standard metabolic equivalents of task (METs) in minutes per day. An equivalent of 3.5 mL O_2_/kg/min (i.e., 71.225 J/kg/min) was used to define 1 MET, and data were summarized as sedentary behavior (≤1.5 METs), light-intensity physical activity (>1.5 and ≤4.0 METs), moderate physical activity (>4 – ≤7 METs) and vigorous physical activity (>7 METs). Physical activity records were included in the analysis if there was a minimum of 48 h of activity recording in weekday and weekend day hours that included at least 12 h from morning (3 am–9 am), noon (9 am–3 pm), afternoon (3 pm–9 pm), and night (9 pm–3 am) to avoid potential bias from over-representing specific times and activities of the days.

*Calculation of total energy expenditure*

As per Pontzer et al. [22], fat-free mass and FM adjusted total energy expenditure (TEE) increases following a power-law, with a rapid increase in neonates and infants up to 1 year old, followed by a decline until ~20 years of age, a stable level between 20 to 60 years and a consecutive decline in older adults. Beyond fat-free mass and FM, male sex was associated with a higher TEE, whereas the pubertal stage was not associated with higher TEE [22].

**Supplementary Results**

*Differences of study cohort according to the study group (intervention versus control)*

At T0, there were no differences in any of the variables based on the study group (intervention or control). At T1 the self-reported time spent on physical activity was higher in the intervention versus control group (123 (42) minutes versus 104 (42) minutes per day, P<0.001). At T2, individuals in the intervention group were older at 15.9 years (0.4) versus 15.7 years (0,4), P=0.017, taller at 1.72 m (0.08) versus 1.69 m (0.09), P=0.017 and reported lower sedentary time at 484 (229) minutes versus 568 (258) minutes per day, P=0.013 compared to the control group. Absolute and proportional intake of dietary proteins, carbohydrates or fats did not differ depending on study group at any age.

**Supplementary References**

1. Lakka, T.A., et al., A 2 year physical activity and dietary intervention attenuates the increase in insulin resistance in a general population of children: the PANIC study. Diabetologia, 2020. 63(11): p. 2270-2281.

2. Eloranta, A.M., et al., The effects of a 2-year physical activity and dietary intervention on plasma lipid concentrations in children: the PANIC Study. Eur J Nutr, 2021. 60(1): p. 425-434.

3. Eloranta, A.M., et al., Dietary factors associated with overweight and body adiposity in Finnish children aged 6-8 years: the PANIC Study. Int J Obes (Lond), 2012. 36(7): p. 950-5.

4. Saari, A., et al., New Finnish growth references for children and adolescents aged 0 to 20 years: Length/height-for-age, weight-for-length/height, and body mass index-for-age. Ann Med, 2011. 43(3): p. 235-48.

5. Tompuri, T.T., et al., Assessment of body composition by dual-energy X-ray absorptiometry, bioimpedance analysis and anthropometrics in children: the Physical Activity and Nutrition in Children study. Clin Physiol Funct Imaging, 2015. 35(1): p. 21-33.

6. Klesges, R.C., et al., Accuracy of self-reports of food intake in obese and normal-weight individuals: effects of parental obesity on reports of children's dietary intake. Am J Clin Nutr, 1988. 48(5): p. 1252-6.

7. Erkkola, M., et al., Nutrient intake variability and number of days needed to assess intake in preschool children. Br J Nutr, 2011. 106(1): p. 130-40.

8. Paturi, M., et al., Picture book of food portion sizes. Helsinki, Finland: Publications of the National Public Health Institute, 2006.

9. Rastas, M., et al., Nutrient composition of foods. 1989.

**Supplementary Table 1, linear regression results for the association between TEI with adiposity measures and TEE**

| BMI z-score at T0, n = 422 | | | | BMI z-score at T1, n = 387 | | | BMI z-score at T2, n = 229 | | |
| --- | --- | --- | --- | --- | --- | --- | --- | --- | --- |
| Predictors | Estimate | 95% CI | P | Estimate | 95% CI | P | Estimate | 95% CI | P |
| Total energy intake (cal) | 0.23 | -0.10 – 0.57 | 0.18 | 0.18 | -0.13 – 0.49 | 0.26 | 0.12 | -0.36 – 0.11 | 0.31 |
| Waist circumference at T0, n = 422 | | | | Waist circumference at T1, n = 387 | | | Waist circumference at T2, n = 229 | | |
| Predictors | Estimate | 95% CI | P | Estimate | 95% CI | P | Estimate | 95% CI | P |
| Total energy intake (cal) | 1.54 | -0.34 – 3.4 | 0.11 | 1.1 | -1.13 – 3.34 | 0.33 | -0.74 | -3.01 – 1.52 | 0.52 |
| Age | 2.53 | 1.10 – 3.97 | <0.001 | 2.94 | 1.29 - 4.60 | <0.001 | 3.75 | 1.03 - 6.47 | <0.001 |
| Sex (male) | 0.78 | -0.37 – 1.94 | 0.18 | 2.20 | 0.65 - 3.74 | 0.005 | 5.89 | 3.41 - 8.36 | 0.007 |
| DXA % Lean mass at T0, n = 414 | | | | DXA % Lean mass at T1, n = 372 | | | DXA % Lean mass at T2, n = 227 | | |
| Total energy intake (cal) | 0.09 | -2.42 – 2.59 | 0.95 | 0.13 | - 1.58 – 4.08 | 0.39 | 4.11 | 2.17 – 6.05 | <0.001 |
| Age | - 2.46 | -4.36 to -0.56 | 0.011 | - 3.17 | - 5.25 to – 1.09 | 0.003 | - 3.00 | -5.33 to - 0.67 | 0.012 |
| Sex (male) | 5.34 | 3.81 - 6.87 | <0.001 | 3.64 | 1.72 - 5.55 | <0.001 | 10.29 | 8.17 - 12.40 | <0.001 |
| DXA % Fat mass at T0, n = 414 | | | | DXA % Fat mass at T1, n = 372 | | | DXA % Fat mass at T2, n = 227 | | |
| Total energy intake (cal) | - 0.12 | -2.70 – 2.44 | 0.92 | - 0.12 | - 4.17 – 1.62 | 0.39 | - 4.30 | - 6.30 to -2.30 | <0.001 |
| Age | 2.48 | 0.54 to 4.43 | 0.012 | 3.19 | 1.06 – 5.33 | 0.003 | 2.99 | 0.59 - 5.39 | 0.015 |
| Sex (male) | - 5.43 | - 6.99 to 3.87 | <0.001 | - 3.69 | - 5.65 to – 1.72 | <0.001 | - 10.19 | -12.37 to - 8.02 | <0.001 |
| TEE at T0, n = 414 | | | | TEE at T1, n = 372 | | | TEE at T2, n = 226 | | |
| Predictors | Estimate | 95% CI | P | Estimate | 95% CI | P | Estimate | 95% CI | P |
| Total energy intake (cal) | 84.70 | 51.15 – 118.26 | <0.001 | 98.31 | 58.41 – 138.21 | <0.001 | 16.33 | 104.0 – 222.6 | 0<0.001 |
| Age | 74.75 | 49.36 - 100.15 | <0.001 | 85.08 | 55.71 – 114.45 | <0.001 | 16.33 | - 0.93 – 141.1 | 0.053 |
| Sex (male) | 115.22 | 94.81 - 135.63 | <0.001 | 109.73 | 82.66 – 136.80 | <0.001 | 524.4 | 459.87 – 589.0 | <0.001 |

**Supplementary Table 2, Mixture models results for adjusted models between proportional macronutrient intake and total energy intake**

| Total energy intake at T0, n = 414 | | | | Total energy intake at T1, n = 370 | | | Total energy intake at T2, n = 226 | | |
| --- | --- | --- | --- | --- | --- | --- | --- | --- | --- |
| Predictors | Estimate | 95% CI | P | Estimate | 95% CI | P | Estimate | 95% CI | P |
| % Energy from Proteins | -1145 | -1958 to -333 | **0.006** | -239 | -1129 – 652 | 0. 60 | -340 | -1413– 732 | 0.53 |
| % Energy from Carbohydrates | 1776 | 1527 – 2025 | **<0.001** | 1699 | 1404 – 1993 | **<0.001** | 1881 | 1506 – 2255 | <0.001 |
| % Energy from Fats | 2884 | 2884 – 32349 | **<0.001** | 2627 | 2258 – 2996 | **<0.001** | 2857 | 2336– 3379 | <0.001 |
| Fiber intake (SD) | 111 | 86– 135 | **<0.001** | 151 | 123– 180 | **<0.001** | 289 | 239 – 339 | <0.001 |
| Physical activity (SD) | 2 | -23 – 28 | 0.87 | 5 | -24 – 34 | 0.73 | -20 | -79 – 39 | 0.50 |
| Sedentary time (SD) | 4 | -19 – 28 | 0.72 | 18 | -9 – 46 | 0.19 | -40 | -91 – 10 | 0.12 |
| Age (SD) | -18 | -44 – 7 | 0.16 | -21 | -50 – 8 | 0.16 | -18 | -68– 32 | 0.49 |
| Total energy expenditure (SD) | 96 | 69 – 122 | **<0.001** | 94 | 65 – 124 | **<0.001** | 238.319 | 185 – 291 | <0.001 |

Adjusted linear mixture model results between % energy from macronutrients in relation to total energy intake. Results at T0, 8 years in left column, at T1, 10 years in middle column and at T2, 16 years in right column. Models adjusted for normalized intake of covariates as indicated in standard deviations for comparison (SD). Abbreviations: N, number of individuals; 95%CI, 95% confidence interval; p, p-value; SD, standard deviation.

**Supplementary Figure 1, Power functions between proportional protein intake and total energy intake, adjusted for sex**

**Supplementary Table 3, Model characteristics of power functions between proportional protein intake and total energy intake additionally adjusted for sex**

| Log energy intake at T0, males, n = 414 | | | |
| --- | --- | --- | --- |
| Predictors | Estimate | 95% CI | P |
| Log % E from Proteins | -0.36 | -0.47 – -0.25 | <0.001 |
| Fiber intake (SD) | 0.07 | 0.05 – 0.08 | <0.001 |
| Physical activity (SD) | -0.00 | -0.02 – 0.02 | 0.896 |
| Sedentary time (SD) | 0.00 | -0.01 – 0.02 | 0.749 |
| Age (SD) | -0.01 | -0.02 – 0.01 | 0.497 |
| Total energy expenditure (SD) | 0.04 | 0.02 – 0.06 | <0.001 |
| Sex (male) | 0.06 | 0.02 – 0.10 | 0.002 |
| Log energy intake at T1, males, n = 370 | | | |
| Predictor | Estimate | 95% CI | P |
| Log % E from Proteins | -0.26 | -0.36 – -0.15 | <0.001 |
| Fiber intake (SD) | 0.09 | 0.07 – 0.11 | <0.001 |
| Physical activity (SD) | -0.00 | -0.02 – 0.02 | 0.934 |
| Sedentary time (SD) | 0.01 | -0.00 – 0.03 | 0.139 |
| Age (SD) | -0.01 | -0.03 – 0.01 | 0.277 |
| Total energy expenditure (SD) | 0.04 | 0.02 – 0.06 | <0.001 |
| Sex (male) | 0.07 | 0.03 – 0.11 | <0.001 |
| Log energy intake at T2, males, n = 226 | | | |
| Predictor | Estimate | 95% CI | P |
| Log % E from Proteins | -0.26 | -0.39 – -0.13 | <0.001 |
| Fiber intake (SD) | 0.15 | 0.12 – 0.18 | <0.001 |
| Physical activity (SD) | -0.03 | -0.06 – 0.00 | 0.090 |
| Sedentary time (SD) | -0.03 | -0.05 – 0.00 | 0.069 |
| Age (SD) | -0.01 | -0.03 – 0.02 | 0.714 |
| Total energy expenditure (SD) | 0.09 | 0.04 – 0.13 | <0.001 |
| Sex (male) | 0.11 | 0.03 – 0.20 | 0.010 |

Adjusted power analysis results for log % Energy from proteins in relation to log total energy intake. Results for males (left column) and females (right column) at To, 8 years in top array, at T1, 10 years in middle array and at T2, 16 years in lowest array. Models adjusted for normalized intake of fiber (in g), for accelerometer-related physical activity and sedentary time (in min), age (in years) and total energy expenditure (in kcal). Abbreviations: N, number of individuals; 95%CI, 95% confidence interval; P, P-value; SD, standard deviation; E, energy in kcal.

**Supplementary Table 4, Model characteristics of adjusted power functions between proportional macronutrient and total energy intake for accelerometery data**

| Log energy intake at T0, n = 373 | | | | | | | | | | | | | |
| --- | --- | --- | --- | --- | --- | --- | --- | --- | --- | --- | --- | --- | --- |
| Predictors | Estimate | 95% CI | P | Predictors | Estimate | | 95% CI | | P | Predictors | Estimate | 95% CI | P |
| Log % E from Proteins | -0.36 | -0.48 to -0.25 | <0.001 | Log % E from Carbohydrates | -0.14 | | -0.33 to -0.04 | | **0.12** | Log % E from Fats | 0.24 | 0.14 – 0.35 | **<0.001** |
| Fiber intake (SD) | 0.07 | 0.05 – 0.09 | <0.001 | Fiber intake (SD) | 0.07 | | 0.05 – 0.09 | | **<0.001** | Fiber intake (SD) | 0.07 | 0.05 – 0.09 | **<0.001** |
| TPA (SD) | 0.10 | -0.39 – 0.59 | 0.70 | TPA (SD) | -0.02 | | -0.54 – 0.49 | | 0.929 | TPA (SD) | -0.01 | -0.51 – 0.49 | 0.96 |
| ST incl. sleep (SD) | 0.08 | -0.41 – 0.57 | 0.74 | ST incl. sleep (SD) | -0.05 | | -0.56 – 0.47 | | 0.860 | ST incl. sleep (SD) | -0.03 | -0.53 – 0.47 | 0.90 |
| Age (SD) | -0.01 | -0.03 – 0.01 | 0.31 | Age (SD) | 0.00 | | -0.02 – 0.01 | | 0.92 | Age (SD) | 0.00 | -0.02 – 0.01 | 0.64 |
| Total energy expenditure (SD) | 0.06 | 0.04 – 0.08 | <0.001 | Total energy expenditure (SD) | 0.06 | | 0.04 – 0.08 | | **<0.001** | Total energy expenditure (SD) | 0.06 | 0.04 – 0.08 | **<0.001** |
| Log energy intake at T1, n = 321 | | | | | | | | | | | | | |
| Predictor | Estimate | 95% CI | P | Predictor | | Estimates | | 95% CI | P | Predictor | Estimates | 95% CI | P |
| Log % E from Proteins | -0.23 | -0.34 to -0.11 | <0.001 | Log % E from Carbohydrates | | -0.19 | | -0.36 to -0.02 | 0.031 | Log % E from Fats | 0.21 | 0.11 – 0.32 | <0.001 |
| Fiber intake (SD) | 0.09 | 0.08 – 0.11 | <0.001 | Fiber intake (SD) | | 0.09 | | 0.0/ – 0.11 | <0.001 | Fiber intake (SD) | 0.09 | 0.08 – 0.11 | <0.001 |
| TPA (SD) | -0.31 | -0.65 – 0.04 | 0. 08 | TPA (SD) | | -0.36 | | -0.71 to -0.01 | 0.04 | TPA (SD) | -0.36 | -0.71 to -0.02 | 0.04 |
| ST incl. sleep (SD) | -0.33 | -0.67 – 0.02 | 0.06 | ST incl. sleep (SD) | | -0.38 | | -0.73 to -0.03 | 0.03 | ST incl. sleep (SD) | -0.38 | -0.73 to -0.04 | 0.03 |
| Age (SD) | -0.01 | -0.03 – 0.01 | 0.26 | Age (SD) | | -0.01 | | -0.03 – 0.01 | 0.34 | Age (SD) | -0.01 | -0.03 – 0.01 | 0.20 |
| Total energy expenditure (SD) | 0.05 | 0.03 – 0.07 | <0.001 | Total energy expenditure (SD) | | 0.05 | | 0.04 – 0.07 | <0.001 | Total energy expenditure (SD) | 0.05 | 0.03 – 0.07 | <0.001 |
| Log energy intake at T2, n = 121 | | | | | | | | | | | | | |
| Predictor | Estimate | 95% CI | P | Predictor | | Estimates | | 95% CI | P | Predictor | Estimates | 95% CI | P |
| Log % E from Proteins | -0.29 | -0.47 to -0.11 | 0.002 | Log % E from Carbohydrates | | -0.12 | | -0.39 - 0.15 | 0.37 | Log % E from Fats | 0.30 | 0.08 – 0.52 | 0.007 |
| Fiber intake (SD) | 0.13 | 0.09 – 0.16 | <0.001 | Fiber intake (SD) | | 0.13 | | 0.09 – 0.16 | <0.001 | Fiber intake (SD) | 0.13 | 0.10 – 0.17 | <0.001 |
| TPA (SD) | -0.87 | -2.53 – 0.80 | 0. 31 | TPA (SD) | | -0.50 | | -2.22 – 1.22 | 0.57 | TPA (SD) | -0.54 | -2.21 – 1.13 | 0.52 |
| ST incl. sleep (SD) | -0.88 | -2.55 – 0.79 | 0.30 | ST incl. sleep (SD) | | -0.50 | | -2.22 – 1.23 | 0.57 | ST incl. sleep (SD) | -0.38 | -2.21 – 1.14 | 0.53 |
| Age (SD) | -0.02 | -0.06 – 0.02 | 0.24 | Age (SD) | | -0.03 | | -0.08 – 0.01 | 0.11 | Age (SD) | -0.04 | -0.08 – 0.00 | 0.07 |
| Total energy expenditure (SD) | 0.13 | 0.09 – 0.17 | <0.001 | Total energy expenditure (SD) | | 0.12 | | 0.08 – 0.16 | <0.001 | Total energy expenditure (SD) | 0.12 | 0.08 – 0.16 | <0.001 |

Adjusted power analysis results for log % Energy from proteins (left column), carbohydrates (middle column) and fats (right column) in relation to log total energy intake. Results at To, 8 years in top array, at T1, 10 years in middle array and at T2, 16 years in lowest array. Models adjusted for normalized intake of fiber (in g), for accelerometer-related physical activity and sedentary time (in min), age (in years) and total energy expenditure (in kcal). Abbreviations: n, number of individuals; 95%CI, 95% confidence interval; P, P-value; SD, standard deviation; E, energy in kcal.
